# Testung auf Mismatch-Reparatur-Defizienz und Mikrosatelliteninstabilität: Eine fokussierte Aktualisierung

**DOI:** 10.1007/s00292-023-01209-1

**Published:** 2023-08-07

**Authors:** Josef Rüschoff, Hans-Ulrich Schildhaus, Jan Hendrik Rüschoff, Korinna Jöhrens, Tina Bocker-Edmonston, Wolfgang Dietmaier, Hendrik Bläker, Gustavo Baretton, David Horst, Manfred Dietel, Arndt Hartmann, Frederick Klauschen, Sabine Merkelbach-Bruse, Albrecht Stenzinger, Sandra Schöniger, Markus Tiemann, Wilko Weichert, Reinhard Büttner

**Affiliations:** 1 Discovery Life Sciences Biomarker GmbH und Pathologie Nordhessen, Germaniastr. 7, 34119 Kassel, Deutschland; 2 Institut für Pathologie und Molekularpathologie, Universitätsspital Zürich, Zürich, Schweiz; 3 Institut für Pathologie, Universitätsklinikum Carl Gustav Carus Dresden, Dresden, Deutschland; 4 Department of Pathology, Cooper University Health Care, Camden, NJ USA; 5 Institut für Pathologie/Zentrum für molekularpathologische Diagnostik, Universität Regensburg, Regensburg, Deutschland; 6 Institut für Pathologie, Universitätsklinikum Leipzig, Leipzig, Deutschland; 7 Institut für Pathologie, Charité – Universitätsmedizin Berlin, Campus Mitte, Berlin, Deutschland; 8 Pathologisches Institut, Universität Erlangen-Nürnberg, Erlangen, Deutschland; 9 Pathologisches Institut, Ludwig-Maximilians-Universität München, München, Deutschland; 10 Institut für Pathologie, Universitätsklinikum Köln, Köln, Deutschland; 11 Pathologisches Institut, Universitätsklinikum Heidelberg, Heidelberg, Deutschland; 12 Institut für Hämatopathologie Hamburg, Hamburg, Deutschland; 13 Institut für Pathologie, Technische Universität München, München, Deutschland

**Keywords:** Hereditäre nonpolypöse kolorektale Neoplasien, Hochdurchsatz-Nukleotidsequenzierung, MMR Immunhistochemie, Immuncheckpoint-Inhibitoren, Lynch-Syndrom, Hereditary nonpolyposis colorectal neoplasms, High-throughput nucleotide sequencing, MMR immunohistochemistry, Immune checkpoint inhibitors, Lynch syndrome

## Abstract

Der Nachweis der Mismatch-Reparatur-Defizienz (dMMR) mit konsekutiver hochgradiger Mikrosatelliteninstabilität (MSI-H) ist inzwischen fester Bestandteil der Diagnostik des kolorektalen Karzinoms (KRK). Galt MSI anfänglich als möglicher Indikator einer erblichen Krebsdisposition (Lynch-Syndrom, LS) steht heute die Vorhersage des Therapieansprechens auf Immuncheckpoint-Inhibitoren (ICI) im Vordergrund. Entsprechende Empfehlungen und Testalgorithmen liegen für den Einsatz in der Primärdiagnostik vor (Übersicht in: Rüschoff et al. 2021).

Aufgrund des damit verbundenen routinemäßigen Einsatzes und des sich erweiternden Indikationsspektrums von ICI-Therapien für Nicht-KRK wie Endometrium‑, Dünndarm‑, Magen- und Gallenwegskarzinome wird eine aktualisierte Übersicht zur dMMR/MSI-Testung vorgelegt. Fokus sind die Herausforderungen bei der Beurteilung immunhistochemischer Färbungen und die Wertigkeit PCR-basierter Verfahren unter Berücksichtigung des erweiterten ICI-Indikationsspektrums. Anhand neuer Daten zur Häufigkeit und Art von Diskordanzen zwischen dMMR- und MSI-Befund und der möglichen Rolle von Next Generation Sequencing zu deren Aufklärung wird ein praxisorientiertes Diagramm zur Entscheidungsfindung im diagnostischen Alltag vorgestellt. Wir weisen zudem auf die Bedeutung systematischer Qualitätssicherungsmaßnahmen (z. B. QuIP MSI-Portal und Ringversuche) einschließlich einer regelmäßigen Fortbildung hin.

**Zusatzmaterial online:**

Die Online-Version dieses Beitrags (10.1007/s00292-023-01209-1) enthält die Tab. S1.

## Abstract (en)

Testing to detect mismatch repair deficiency (dMMR) and high-grade microsatellite instability (MSI-H) has become an integral part of the routine diagnostic workup for colorectal cancer (CRC). While MSI was initially considered to be a possible indicator of a hereditary disposition to cancer (Lynch syndrome, LS), today the prediction of the therapy response to immune checkpoint inhibitors (ICI) is in the foreground. Corresponding recommendations and testing algorithms are available for use in primary diagnosis (reviewed in: Rüschoff et al. 2021).

Given the increasing importance for routine use and the expanding indication spectrum of ICI therapies for non-CRCs, such as endometrial, small intestinal, gastric, and biliary tract cancers, an updated review of dMMR/MSI testing is presented. The focus is on the challenges in the assessment of immunohistochemical stains and the value of PCR-based procedures, considering the expanded ICI indication spectrum. A practice-oriented flowchart for everyday diagnostic decision-making is provided that considers new data on the frequency and type of discordances between MMR-IHC and MSI-PCR findings, and the possible role of Next Generation Sequencing in clarifying them. Reference is made to the significance of systematic quality assurance measures (e.g., QuIP MSI portal and multicenter proficiency testing), including regular continued training and education.

Der Verlust der Fähigkeit einer Zelle zur Reparatur von Fehlpaarungen (Mismatches) in einfach repetitiven (Mikrosatelliten‑)DNA-Abschnitten wird in erster Linie durch biallelische Inaktivierung der Mismatch-Reparatur(MMR-)Proteine MLH1, MSH2, MSH6 und PMS2 verursacht. In den meisten Fällen (70–80 %) ist MLH1 aufgrund einer mit dem Alter zunehmenden Methylierung Cytidin-reicher Promotorabschnitte betroffen (erworbene Form). Ferner treten pathogene MMR-Genmutationen auf, die meist über die Keimbahn vererbt werden (hereditäre/konstitutionelle Form) und seltener auch somatisch erworben werden können (Review in Schöniger, Rüschoff [25]).

Über viele Jahre empfahl man die Untersuchung auf Mismatch-Reparatur-Defizienz (dMMR) mit konsekutiver hochgradiger Mikrosatelliteninstabilität (MSI-H) primär bei auffälliger Familienanamnese als Hinweis auf eine mögliche erbliche Tumordisposition aus dem Formenkreis des Lynch-Syndroms (LS) speziell im kolorektalen Karzinom (KRK) und Endometriumkarzinom (EK). Mit dem Nachweis einer hohen Ansprechrate von Tumoren vom dMMR-/MSI-H-Typ auf Immuncheckpoint-Inhibitoren (ICI) [14] hat inzwischen die Empfehlung zur Testung aller KRK und EK bereits bei Primärdiagnose Eingang in die entsprechenden Therapieempfehlungen auf nationaler und internationaler Ebene gefunden ([2]; Übersicht in Rüschoff et al. [22]). Seitens der Europäischen Arzneimittel-Agentur (EMA) erfolgte 2021 die Zulassung zweier PD-1-gerichteter ICI mit Pembrolizumab in der Erstlinie für das metastasierte KRK [1] und Dostarlimab in der Zweitlinie bei Rezidiv oder Therapieversagen bei EK [21]. Anfang 2022 erfolgte eine Indikationserweiterung für Pembrolizumab auf nichtresezierbare oder metastasierte Endometrium‑, Magen- und Dünndarmkarzinome sowie biliäre Karzinome mit MSI-H- oder dMMR-Status [19]. Damit stellt sich die Frage, inwieweit die langjährigen Erfahrungen der dMMR- und MSI-Testung des KRK (Übersicht: [5]) auf die neuen Indikationen übertragen werden können und worin ggf. die zu berücksichtigenden Unterschiede liegen.

Auch aufgrund der Methodenverfügbarkeit gehört die immunhistochemische Testung auf dMMR inzwischen zum Standardrepertoire der Pathologie, wobei dies aufgrund vereinfachter Technologieplattformen [24, 30] zunehmend auch für die Testung auf MSI gilt. Die aktuellen Empfehlungen empfehlen beide als bevorzugte Verfahren zur Diagnostik, wobei die ESMO-Guidelines die Immunhistochemie (IHC) als die zuerst einzusetzende Testmethode vorschlagen [16, 34]. Aufgrund des inzwischen deutlich erweiterten Indikationsspektrums der ICI-Therapien werden in der hier vorgelegten fokussierten Übersicht ausgehend vom KRK die Erfahrungen mit der dMMR- und MSI-Testung kritisch beleuchtet und neu bewertet.

## Wertigkeit von MMR-IHC und MSI-PCR in unterschiedlichen Organsystemen

In der aktuellen ASCO/CAP-Richtlinie [4] werden 6 Empfehlungen ausgesprochen, wovon 4 bereits die unterschiedliche Wertigkeit der Methoden je nach Organsystem berücksichtigen. Beim KRK sind MMR-IHC und MSI-PCR gleichwertig. Next Generation Sequencing (NGS) kann eingesetzt werden, wenn es gegen diese Testverfahren validiert worden ist (Empfehlung 1). Beim Adenokarzinom am ösophagogastralen Übergang und des Dünndarms sind MMR-IHC und MSI-PCR dem NGS-Verfahren überlegen (Empfehlung 2). Beim Endometriumkarzinom ist die Immunhistochemie beiden DNA-basierten Verfahren überlegen (Empfehlung 3). Für alle übrigen Tumorentitäten lagen zum Zeitpunkt der zugrunde liegenden Literaturrecherche (bis 02/2020) keine ausreichenden Literaturdaten vor, sodass auch hier der Immunhistochemie – bis zum Vorliegen weiterer Evidenz – der Vorzug gegeben werden sollte (Empfehlung 4).

Eine aktuelle umfangreiche Analyse einer französischen Arbeitsgruppe von insgesamt 3800 Tumoren, die jeweils parallel mittels MMR-IHC und MSI-PCR über 10 Jahre untersucht wurden [10], ergibt einen praxisrelevanten Ansatz. Sie diagnostizierten 15,4 % (*n* = 585) der Fälle als dMMR und/oder MSI‑H. Die möglichen Befundkonstellationen aus MMR- und MSI-Analyse unterteilten sie in *klassische Befunde* (84,7 %, *n* = 496) mit Ausfall der jeweiligen Heterodimerisierungsproteine MLH1/PMS2 oder MSH2/MSH6 und davon abweichende *ungewöhnliche Befunde* (15,2 %, *n* = 89). Letztere umfassten 4 Gruppen mit unterschiedlicher Befundkonstellation:

1. isolierter Ausfall von PMS2 oder MSH6,
2. Ausfall beider Bindungspartner, aber kein MSI‑H,
3. erhaltene MMR-Expression, aber MSI-H/MSI‑L und
4. komplexe immunhistochemische Befunde mit z. B. nur herdförmigem (klonalem) Expressionsverlust, Ausfall mehrerer MMR-Proteine oder von MSH2 mit PMS2-Ausfall.

**Figure 1:**
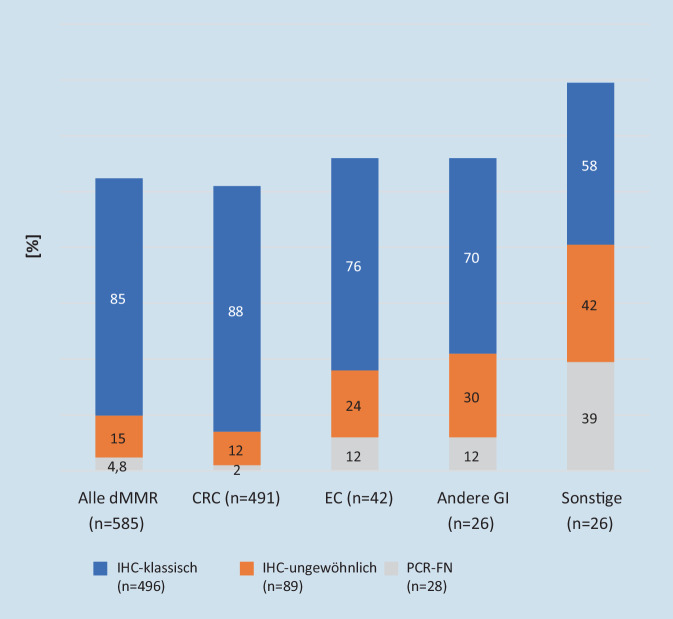

Es zeigte sich, dass diese ungewöhnlichen Befundkonstellationen gerade nichtkolorektale Neoplasien betreffen und mit einer höheren Wahrscheinlichkeit eines falsch negativen PCR-Ergebnisses einhergehen (Abb. 1).

Bemerkenswerterweise ließen sich Fälle mit ungewöhnlicher Befundkonstellation etwa doppelt so häufig auf ein Lynch-Syndrom zurückführen wie dies bei klassischem Befund der Fall war (42,7 % vs. 21,4 %). Von 26 mittels NGS (FoundationOne®-Test, Roche, Basel, Schweiz) untersuchten Tumoren mit ungewöhnlicher Befundkonstellation waren die Mehrzahl MSI‑H (85 %) und zeigten eine erhöhte Tumormutationslast (21 × TMB-high, 4 × TMB-intermediate). Die Autoren folgern, dass Tumoren mit ungewöhnlicher Befundkonstellation in der MMR-IHC nicht von vornherein von einer ICI-Therapie ausgeschlossen werden sollten und dass letztlich die dMMR-Analyse nur wenige, potenziell für diese Therapie geeignete Patienten (< 1 %) verpasst.

Nachfolgend wird die von unserer Gruppe vorgelegte Empfehlung einer schrittweisen, initial auf 2 MMR-Antikörpern (PMS2, MSH6) basierenden MMR/MSI-Diagnostik [22] unter besonderer Berücksichtigung neuer Daten und Empfehlungen aktualisiert [4, 10, 20, 31]. Dabei liegt der Fokus auf den Herausforderungen der MMR-/MSI-Testung im diagnostischen Alltag, die sich aus dem erweiterten Indikationsspektrum der ICI-Therapie ergeben, und mündet im Vorschlag eines optimierten Testalgorithmus.

## Klassischer dMMR-Befund

Der typische Fall von dMMR ist der komplette Ausfall der Immunreaktion für eines der beiden MMR-Protein-Heterodimere (MLH1/PMS2 oder MSH2/MSH6) und im französischen Datensatz [10] mit ca. 85 % der MSI-H/dMMR-Befunde die mit Abstand größte Gruppe. Dieser Befund kennzeichnet mit großer Sicherheit eine Neoplasie vom MSI-Typ, in 96,9 % (496/512) lag ein MSI-H-Status vor. Die Übereinstimmung von dMMR und MSI‑H war mit 98,8 % (485/491) beim KRK am größten, gefolgt von den nichtkolorektalen Gastrointestinal(GI)- (92,9 %) und den Endometriumkarzinomen (91,4 %). In der Gruppe der sonstigen Tumorentitäten lag der Anteil konkordanter dMMR- und MSI-H-Befunde nur noch bei 79 %. Außerhalb des Kolons ist demnach die PCR weniger sensitiv und in der Praxis empfiehlt sich eine schrittweise Vorgehensweise mit initialer MMR-IHC ([4, 31], Übersicht in Rüschoff et al. [22]).

## Ungewöhnlicher dMMR-Befund

Hierunter werden alle Befunde subsumiert, die vom zuvor beschriebenen klassischen IHC-Befund mit Komplettausfall der MMR-Bindungspartner abweichen.

### Isolierter Ausfall von PMS2 oder MSH6

Der isolierte Ausfall von PMS2 oder MSH6 als jeweiligem Heterodimerisierungspartner von MLH1 bzw. MSH2 bildete die Mehrzahl aller untypischen Befunde (53/89) mit einer Prävalenz von etwa 8 % bei dMMR-KRK, 10 % beim EK und je 19 % bei den übrigen GI-Tumoren und sonstigen Tumoren. In der MSI-PCR erwiesen sich 81,1 % (43/53) als MSI‑H. In 36 Fällen wurde eine weitergehende genetische Untersuchung angeschlossen. Für fast die Hälfte dieser Fallgruppe (45,3 %) ergab sich ein genetischer Hintergrund: 10 × PMS2 und 12 × MSH6 assoziiertes Lynch-Syndrom (LS) sowie je 1 × POLE-assoziierte und konstitutionelle MMR-Defizienz (CMMRD). Bemerkenswerterweise waren 20 % (5/24) der Fälle mit nachgewiesenem erblichen Tumorsyndrom und isoliertem PMS2- bzw. MSH6-Verlust in der PCR stabil (MSS).

#### Molekularer Hintergrund.

In dieser Gruppe werden MMR-Proteine zusammengefasst, die isoliert betroffen in erster Linie durch Keimbahnmutationen ausgeschaltet werden und somit im Zusammenhang mit dem LS stehen [20].

Es ist bekannt, dass Keimbahnmutationen von *MSH6* und *PMS2* gegenüber *MLH1* und *MSH2* eine deutlich geringere Penetranz mit niedrigerem Krebslebenszeitrisiko aufweisen, wobei Keimbahnmutationen im *MSH6*-Gen speziell für Frauen das Risiko eines EK erhöhen [6, 20, 32].

Dementsprechend ist die MSI-Ausprägung bei isoliertem MSH6- oder PMS2-Ausfall geringer mit meist nur diskreten Shifts in der MSI-PCR, die leicht übersehen werden können. So zeigten in der Studie von Stelloo et al. [29] mit 696 EK nur die Hälfte der Fälle mit isoliertem MSH6-Ausfall (*n* = 10) einen MSI-H-Phänotyp (Promega®-System, Promega, Fitchburg, WI, USA). In einer weiteren Methodenvergleichsstudie erwies sich beim EK die IHC den PCR-Methoden einschließlich NGS überlegen. Der Tumorgehalt hat sich als kritisch für das PCR-Ergebnis herausgestellt. Er sollte im Idylla®-System (Biocartis NV, Mechelen, Belgien) anstatt bei 20 % (für KRK) bei mindestens 40 % liegen [27].

#### Empfehlung.

**Figure 2:**
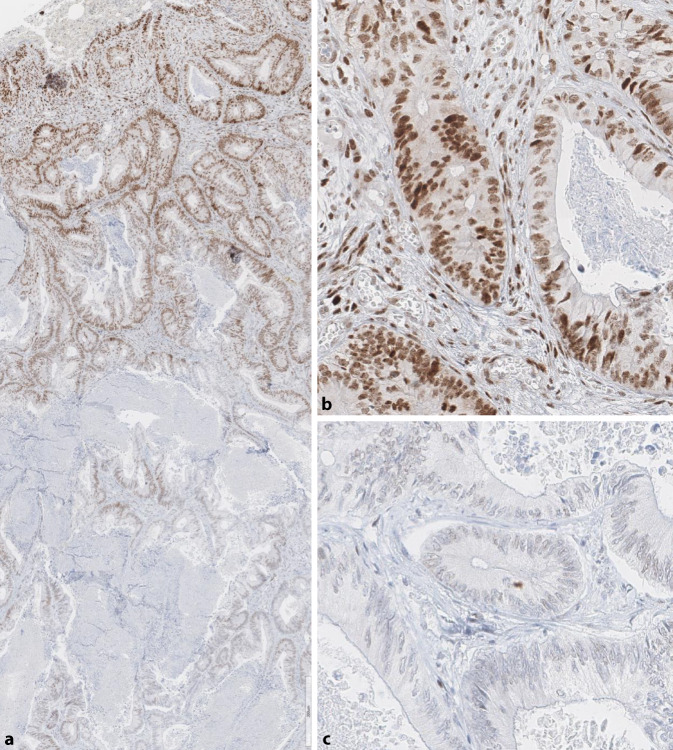

Grundsätzlich sollte bei isoliertem PMS2-Ausfall zunächst eine Fehlinterpretation des immunhistochemischen Befundes, z. B. aufgrund eines Färbegradienten, und insbesondere ein sog. punktiertes Färbemuster bei MLH1 ausgeschlossen werden (Abb. 2 und 4). Um eine Beteiligung von MLH1 sicher auszuschließen, ist ggf. auch eine zusätzliche *BRAF*- (bei KRK) und/oder Promotormethylierungsanalyse bei anderen Tumorentitäten in Betracht zu ziehen [20, 33]. Ein isolierter Ausfall der MSH6-Immunreaktion ist beim Rektumkarzinom nach Radiochemotherapie beschrieben [3, 8], wobei dies typischerweise nicht mit MSI‑H einhergeht. Ein Patient mit *MSH6*-bedingtem LS und vorbehandeltem Rektumkarzinom im französischen Kollektiv war dagegen MSI‑H [10].

Grundsätzlich wird in Fällen eines isolierten PMS2- und MSH6-Ausfalls die Überprüfung des Befundes mittels PCR empfohlen. Allerdings schließt ein MSS- oder MSI-L-Status ein syndromales Geschehen nicht aus. Auch diese Fälle sollten klinisch (Anamnese) und humangenetisch abgeklärt werden. Tumore mit zusätzlich positiver PCR (MSI-H) und wahrscheinlich auch bei syndromalem Hintergrund und Mutationsnachweis sind für eine ICI-Therapie geeignet. Inwieweit dies bei fehlender MSI-H-Konstellation und alleinigem immunhistochemischem dMMR-Nachweis ebenso der Fall ist, ist bislang nicht abschließend geklärt. Solche Konstellationen könnten Ursache von Therapierresistenz sein [31]. In dem Kollektiv von Jaffrelot et al. [10] betraf dies immerhin 18,8 % (10/53) der Fälle. Zu bedenken ist ggf. eine NGS-Analyse mit der sowohl neben dem MSI- und TMB-Status (TMB, „tumor mutational burden“, Tumormutationslast) insbesondere auch der zugrunde liegende Mutationsstatus bestimmt werden können. Entsprechend wurden 3 der 10 Fälle weiter untersucht. Es zeigte sich je 1 × eine *PMS2*- bzw. *MSH6-*Keimbahnmutation, 1 × eine *POLE*-Mutation mit offenbar sekundärem (somatischem) MSH6-Ausfall. Letzteres könnte als Hinweis auf Wirksamkeit einer ICI-Therapie herangezogen werden [17]. Ein weiterer Fall mit isoliertem PMS2-Ausfall zeigte eine konstitutionelle, biallelische *PMS2*-Keimbahnmutation (CMMRD). Diese fallen immunhistochemisch durch Expressionsverlust in Tumor- und Normalgewebe auf [13] und können in der PCR übersehen werden [30]. Sie sind somit auch für ICI-Therapie geeignet. In jedem Fall wird eine entsprechende Beschreibung im diagnostischen Befundbericht empfohlen [10].

### Diskordanz zwischen MMR-IHC und MSI-PCR

In der Literatur werden widersprüchliche Befunde zwischen IHC und PCR als diskordant bezeichnet, wenn entweder trotz Expressionsverlust eines MMR-Proteins die MSI-PCR einen stabilen Phänotyp (MSS/MSI-L) zeigt oder umgekehrt sich trotz MSI-H-Befund in der MMR-IHC kein Proteinausfall findet (pMMR).

Die Konstellation dMMR ohne MSI-H-Nachweis trat mit 1,2 % (6/491) am seltensten im KRK auf, war bei nichtkolorektalen GI-Tumoren mit 7,1 % (2/28) und beim EK mit 8,6 % (4/46) deutlich häufiger zu beobachten, am häufigsten aber mit 21 % (4/19) bei den sonstigen Tumorentitäten [10]. Dies bestätigen auch Daten einer aktuellen Untersuchung an 4 LS-Patienten (2 × *MSH2*-, 2 × *MSH6*-Keimbahnmutation) [15] mit Mehrfachtumoren und/oder Metastasen. Die Immunhistochemie ergab in allen Primärkarzinomen (2 × Kolon, 2 × Rektum) und 7 anderen Tumoren (klarzelliges EK mit Metastase, Urothelkarzinom, Nebennierenrindenkarzinom mit Metastase, 2 × Sarkome) den klassischen MMR-Befund bei *MSH2*- und einen isolierten MSH6-Ausfall bei *MSH6*-Mutationsträgern. Die MSI-Analyse mit dem Bethesda- und Promega®-Mononukleotidpanel ergab einen MSI-H-Status in allen 4 KRK, aber nur in einem der anderen 7 Tumoren, was einer Diskordanzrate von 86 % im extrakolischen Bereich bei diesen LS-Patienten entspricht.

#### Empfehlung.

**Figure 3:**
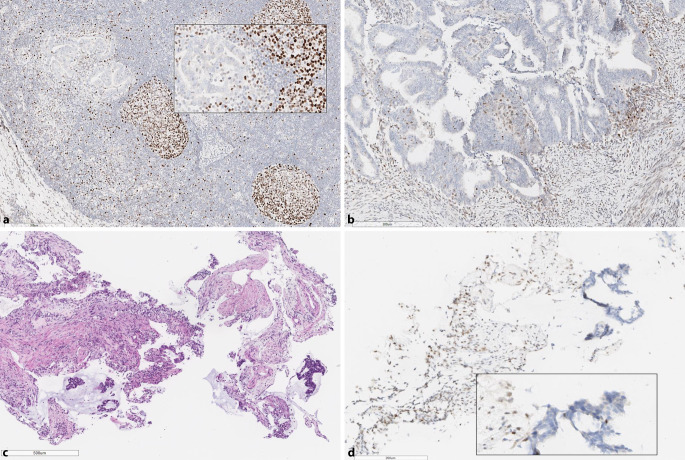

Beim Befund (dMMR/MSS) sollten zunächst probenbedingte Aspekte wie der Gehalt an Tumorzellen im PCR-Probenansatz überprüft werden (Abb. 3)**.** Darüber hinaus sind tumorbiologische Aspekte zu berücksichtigen. So nimmt der Grad der Instabilität im Laufe der Tumorprogression zu [12] und kann in frühen pT1-Tumoren zu falsch negativen PCR-Befunden führen. Schließlich sind auch die genannten Unterschiede zwischen den *MMR*-Genen zu berücksichtigen mit eher geringer ausgeprägtem MSI-Phänotyp und nur diskreten Shifts bei *PMS2*- und *MSH6*-Mutationen. Diese können z. B. auch bei Verzicht auf normales Kontrollgewebe in der PCR (z. B. beim Idylla®-System) übersehen werden [27]. Teilweise sind über sekundäre *MSH3*-Mutationen weniger die Mono-, sondern eher die längeren Di- und Trinukleotidrepeats betroffen, sodass auch dieser MSI-Phänotyp mononukleotidbasierten Testverfahren (Promega®, Idylla®) entgehen kann; hier sollte sicherheitshalber das Bethesdapanel angeschlossen werden [5].

Unter der Maßgabe, dass die Immunhistochemie als initiale Screeningmethode eingesetzt wird, ist die umgekehrte Befundkonstellation mit eindeutigem MSI-H-Befund und erhaltener MMR-Proteinexpression (pMMR vs. MSI-H) von besonderer Bedeutung.

In dem großen französischen Untersuchungskollektiv erfüllten nur 3 Fälle diese Form der Diskordanz, allesamt KRK. Zweimal konnte ein LS mit *MSH2*- und *PMS2*-Mutation nachgewiesen werden, einmal wurde mittels NGS eine somatische Doppelmutation im *MLH1*- und *PMS2*-Gen gesichert. Eine frühere Arbeit aus der Arbeitsgruppe von Shia gab die Diskordanzrate mit ca. 6 % der KRK an [9]. Allerdings wurden in dieser Arbeit die ungewöhnlichen IHC-Befunde noch der Gruppe mit erhaltener MMR-Expression zugerechnet. Dies wurde in der aktuellen Arbeit derselben Gruppe inzwischen aufgegeben [31]. Jegliche Abweichung von kompletter klassischer Expression (pMMR) wird nun als abnormal interpretiert, was die deutlich niedrigere Diskordanzrate der französischen Daten erklärt [10].

#### Empfehlung.

**Figure 4:**
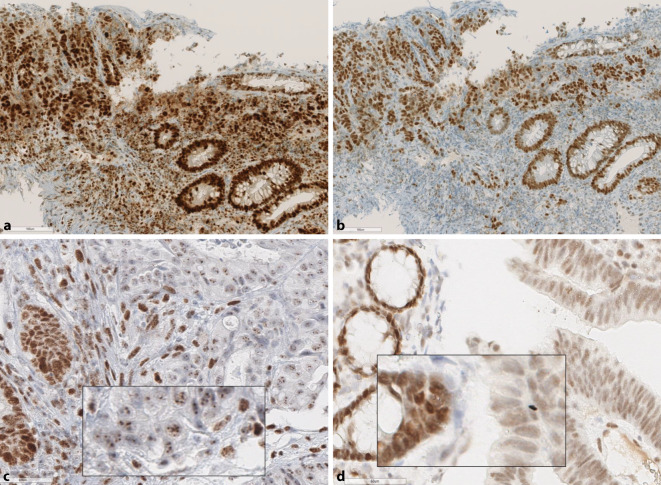

Zur bestmöglichen Vermeidung eines falsch negativen MMR-IHC-Befundes sollten bei jedem Fall mit komplett erhaltener MMR-Proteinexpression immer auch die Bethesdakriterien, v. a. das Patientenalter, beachtet werden. Bei jüngeren Patienten (< 60 J.) empfiehlt sich die PCR-Analyse und ggf. eine weitergehende humangenetische Beratung bzw. NGS-Analyse zur Abklärung des Mutationsstatus (Abb. 4).

### Komplexe Befundkonstellationen

**Figure 5:**
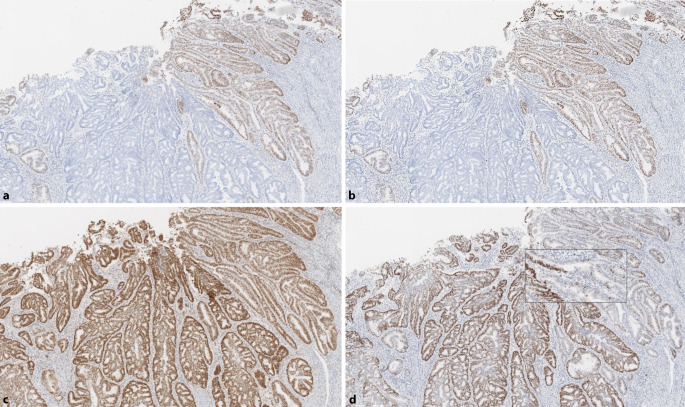

Eine in der Alltagsroutine schwierige Befundgruppe bilden die Tumoren, bei denen z. B. mehr als nur die 2 typischen MMR-Bindungspartner ausgefallen sind (in der französischen Studie: 3 × 3 MMR- und 4 × alle 4 MMR-Proteine). Besonders herausfordernd ist die Beurteilung von Färbeabschwächungen, die sich immer auf die interne Gewebekontrolle beziehen und hohe Qualitätsstandards mit gut fixiertem Probenmaterial erfordern (Abb. 4d). Dies gilt auch für die Interpretation von klonalen Färbeausfällen, von denen in der Regel gesprochen wird, wenn diese mindestens 10 % eines Tumors betreffen (Abb. 5). Diese Befunde – in der ASCO/CAP Guideline [4] und im WHO Band zum KRK [8] noch als nicht pathologisch ohne Bezug zum LS bezeichnet – werden inzwischen als abnormal interpretiert [31]. Auch die französischen Daten zeigen gerade bei solchen „komplexen“ Färbebefunden in mehr als der Hälfte (53,3 %) einen Bezug zum LS. Zumeist fand sich ein MSI-H-Status (13/15), sodass sich zumindest diese Fälle für ICI-Therapie eignen.

#### Empfehlung.

Grundsätzlich setzt die Diagnose eines komplexen immunhistochemischen MMR-IHC-Befundes eine validierte und präzise eingestellte immunhistochemische Untersuchungstechnik voraus. Areale mit fehlender oder abgeschwächter interner Färbekontrolle sind von der Befundung auszuschließen. Bei klonalem Ausfall empfiehlt sich für die PCR-Analyse eine an der MMR-IHC orientierte Mikrodissektion (Abb. 5). Der Befundbericht sollte den Sachverhalt analog Tab. 1 widerspiegeln mit entsprechender Empfehlung zu einer weitergehenden humangenetischen Untersuchung oder auch NGS zur Abklärung des Mutationsstatus [20, 23, 31].

**Figure 6:**
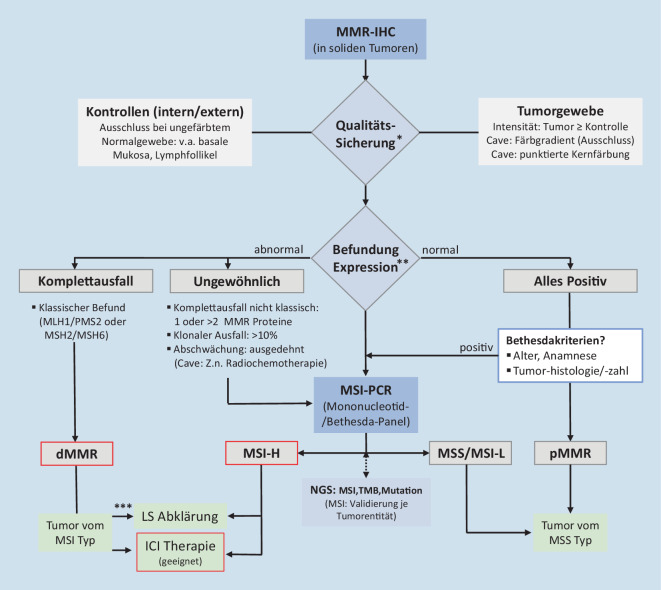

**Table 1:** 

*Färbeabschwächungen*	*TU: int. CTRL*	*Interpretation*
MLH1/PMS2	< / ~	Normal
MLH1/PMS2	< / *n*	Beide abnormal
MLH1/PMS2	~ / <	PMS2 fraglich
MLH1/PMS2	*n* / <	Beide abnormal
MSH2/MSH6	< oder *n* / ~	MSH2 fraglich; ggf. sekundär somatisch bei *POLE*-Mutation
MSH2/MSH6	< / *n*	Beide abnormal
MSH2/MSH6	~ / <	MSH6 abnormal
MSH2/MSH6	*n* / <	Beide abnormal
*Klonaler Verlust*	*Abrupter Ausfall*	*Interpretation*
MLH1/PMS2	Beide	Klonale MLH1-Methylierung od. Keimbahnmutation (Hum.Gen.)
MSH6 bei MLH1/PMS2-Mismatch-Repair-Defizienz	Nur MSH6	Sekundäre Mutation in codierender *MSH6*-Region (C8-Repeat)
MLH1/PMS2, MSH2/MSH6 oder PMS2 oder MSH6 allein	Beide oder allein	Kann genetisch bedingt sein (Hum. Gen.)
*Mehrfacher Komplettausfall*	*Bei pos int. CTRL*	*Interpretation*
MLH1/PMS2 u. MSH6	3 Proteine	Sekundäre Mutation in codierender *MSH6*-Region (C8-Repeat)
MLH1/PMS2 u. MSH2/MSH6	4 Proteine	Sekundäre Mutation in intronischem *MSH2*-Repeat (BAT25)

## Update schrittweise Beurteilung MMR-IHC

Die Indikationsausweitung von ICI-Therapien auf Nicht-KRK macht eine Neubeurteilung und Anpassung der Testalgorithmen erforderlich. Neue Studiendaten belegen die Überlegenheit der MMR-IHC gegenüber der MSI-PCR in diesen Indikationen. Molekulare Methoden sollten bei nicht eindeutigen MMR-IHC-Befunden zur weiteren Abklärung eingesetzt werden. Bei diskordanter Befundkonstellation können die Untersuchung der MLH1-Promotormethylierung (*BRAF*-Mutation beim KRK) [33] und die NGS-Analyse zur Abklärung des Mutationsstatus von Nutzen sein. Eine NGS Analyse zur Bestimmung des Mikrosatellitenstatus sollte für das jeweilige Organsystem gegen MMR-IHC und MSI-PCR validiert werden [10, 11, 28].

Für eine verlässliche MMR-IHC ist eine hochwertige Qualitätssicherung mit Teilnahme an Ringversuchen und Weiterbildungen (z. B. QuIP, QuIP-Portal) erforderlich. Der Prozess der MMR-Analytik beginnt mit der optimalen Fixierung des zu untersuchenden Gewebes und der Beachtung präanalytischer Faktoren [18]. Für eine Erfassung diagnostisch relevanter Färbeabschwächungen sind optimal fixierte Proben essenziell, weshalb, wenn möglich, Biopsien Resektaten vorzuziehen sind [7, 22]. Erforderlich sind optimale, v. a. interne Färbekontrollen mit Nachweis einer guten bis kräftigen Immunreaktion im Normalgewebe. Proben mit nur schwach angefärbten Lymphfollikelzentren oder Epithelzellen am Kryptengrund sollten im Zweifel neu gefärbt oder das Färbeprotokoll angepasst werden. In Ringversuchen haben sich bestimmte Antikörperklone als besonders robust erwiesen (s. Liste in [31] Supplement). Ventana hat ein Antikörperpanel mit FDA-Zulassung zusammengestellt.

Während bislang vor allem nur der komplette Ausfall der Immunreaktion als typisch für dMMR gewertet wurde [4, 8], schlagen Wang et al. [31] vor, nur die Fälle mit „all present“ als *normal* und alle anderen als *abnormal *anzusehen, was neben den typischen Befunden (Komplettausfall) auch die mit teilweisem Ausfall oder Abschwächung miteinbezieht. Diesem Vorgehen entspricht der Ansatz der französischen Arbeitsgruppe [10], bei dem alle in der MMR-IHC nicht vollständig positiven oder negativen Tumoren als „ungewöhnlich“ klassifiziert wurden. In dieser Gruppe kommen besonders häufig (bis 50 %) erbliche Syndrome (meist LS, selten POLE oder CMMRD) vor [10]. Fälle mit komplettem MMR-Proteinausfall und MSI‑H sind potenziell für eine ICI-Therapie geeignet. Wie Tumoren mit z. B. klonalem MMR-Verlust ohne MSI‑H auf ICI-Therapie ansprechen, ist bisher ungeklärt. Zur Klärung sind weitergehende Studien erforderlich.

## Fazit für die Praxis

- Im kolorektalen Karzinom (KRK) stimmen immunhistochemische Mismatch-Reparatur-Defizienz (dMMR) und PCR-basierte Mikrosatelliteninstabilitäts(MSI)-Analysen in hohem Maße (> 98 %) überein. In anderen Gastrointestinaltumoren und Endometriumkarzinomen treten in ca. 5–10 %, in sonstigen Tumoren in bis zu 40 % Diskordanzen auf.
- Bei Nicht-KRK ist die Mismatch-Repair-Immunhistochemie (MMR-IHC) der MSI-PCR überlegen, sofern stringente Qualitätskriterien (Antikörperwahl, Färbeprotokoll und Auswertung) eingehalten werden. Für alle Methoden ist eine optimale Fixierung essenziel.
- Traditionell bezieht sich die Definition von dMMR auf den Komplettausfall eines MMR-Proteins. Heute wird empfohlen, die komplett positive Reaktion als normal (pMMR, Mismatch-Repair-Profzienz) anzusehen und somit jegliche Abweichung (kompletter oder partieller Verlust) als dMMR (abnormal) zu klassifizieren.
- In der Praxis sollte der IHC-Befund in normal (alle MMR-Proteine positiv) und abnormal mit klassischer dMMR (Komplettausfall zweier Bindungspartner) oder davon abweichende, ungewöhnliche Befunde eingeteilt werden. Letztere sind besonders häufig Lynch-Syndrom-assoziiert und können durch molekulare Methoden abgesichert werden.
- Tumoren mit klassischer dMMR und/oder hochgradiger Mikrosatelliteninstabilität (MSI-H) sind für Immuncheckpoint-Inhibitor(ICI)-Therapie geeignet. Bei reduzierter/heterogener MMR-Proteinexpression ohne MSI‑H besteht Unsicherheit. Hier sind eingehender Probenabgleich und ggf. weitergehende molekulare Untersuchung (z. B. Next Generation Sequencing) erforderlich.

## Supplementary Information





## References

[1] André T, KEYNOTE-177 Investigators (2020). Pembrolizumab in microsatellite-instability-high advanced colorectal cancer. N Engl J Med.

[2] AWMF (2022). S3-Leitlinie Endometriumkarzinom.

[3] Bao F (2010). Neoadjuvant therapy induces loss of MSH6 expression in colorectal carcinoma. Am J Surg Pathol.

[4] Bartley AN, Mills AM, Konnick E (2022). Mismatch repair and microsatellite instability testing for immune checkpoint inhibitor therapy: guideline from the college of American pathologists in collaboration with the association for molecular pathology and fight colorectal cancer. Arch Pathol Lab Med.

[5] Dietmaier W, Büttner R, Rüschoff J (2019). Mikrosatelliteninstabilität: Aktueller Überblick über Methoden und Anwendungen. Pathologe.

[6] Dominguez-Valentin M (2020). Cancer risks by gene, age, and gender in 6350 carriers of pathogenic mismatch repair variants. Genet Med.

[7] Fadhil W, Ilyas M (2012). Immunostaining for mismatch repair (MMR) protein expression in colorectal cancer is better and easier to interpret when performed on diagnostic biopsies. Histopathology.

[8] Frankel WL (2019). Lynch Syndrome.

[9] Hechtman JF, Rana S, Middha S (2020). Retained mismatch repair protein expression occurs in approximately 6 % of microsatellite instability-high cancers and is associated with missense mutations in mismatch repair genes. Mod Pathol.

[10] Jaffrelot M, Farés N, Brunac AC (2022). An unusual phenotype occurs in 15 % of mismatch repair-deficient tumors and is associated with non-colorectal cancers and genetic syndromes. Mod Pathol.

[11] Kang SY, Kim DG, Ahn S (2022). Comparative analysis of microsatellite instability by next-generation sequencing, MSI PCR and MMR immunohistochemistry in 1942 solid cancers. Pathol Res Pract.

[12] Kloth M, Ruesseler V, Engel C (2016). Activating ERBB2/HER2 mutations indicate susceptibility to pan-HER inhibitors in Lynch and Lynch-like colorectal cancer. Gut.

[13] Krüger S, Kinzel M, Walldorf C (2008). Homozygous PMS2 germline mutations in two families with early-onset haematological malignancy, brain tumours, HNPCC-associated tumours, and signs of neurofibromatosis type 1. Eur J Hum Genet.

[14] Le DT, Uram JN, Wang H (2015). PD-1 blockade in tumors with mismatch-repair deficiency. N Engl J Med.

[15] Li Z, Cheng B, Liu S (2022). Non-classical phenotypes of mismatch repair deficiency and microsatellite instability in primary and metastatic tumors at different sites in Lynch syndrome. Front Oncol.

[16] Luchini C, Bibeau F, Ligtenberg MJL (2019). ESMO recommendations on microsatellite instability testing for immunotherapy in cancer, and its relationship with PD-1/PD-L1 expression and tumour mutational burden: a systematic review-based approach. Ann Oncol.

[17] Ma X, Dong L, Liu X, Ou K, Yang L (2022). POLE/POLD1 mutation and tumor immunotherapy. J Exp Clin Cancer Res.

[18] Malapelle U, Parente P, Pepe F (2020). Impact of pre-analytical factors on MSI test accuracy in mucinous colorectal adenocarcinoma: a multi-assay concordance study. Cells.

[19] Marabelle A, Le DT, Ascierto PA (2020). Efficacy of pembrolizumab in patients with noncolorectal high microsatellite instability/mismatch repair-deficient cancer: results from the phase II KEYNOTE-158 study. J Clin Oncol.

[20] NCCN (2022) https://www.nccn.org/professionals/physician_gls/pdf/genetics_colon.pdf. Zugegriffen: 3. März 2023

[21] Oaknin A, Gilbert L, Tinker AV (2020). LBA36—safety and antitumor activity of dostarlimab in patients (pts) with advanced or recurrent DNA mismatch repair deficient (dMMR) or proficient (MMRp) endometrial cancer (EC): results from Garnet. Ann Oncol.

[22] Rüschoff J, Baretton G, Bläker H (2021). MSI-Testung : Was ist neu? Was ist zu beachten?. Pathologe.

[23] Salem ME, Bodor JN, Puccini A (2020). Relationship between MLH1, PMS2, MSH2 and MSH6 gene-specific alterations and tumor mutational burden in 1057 microsatellite instability-high solid tumors. Int J Cancer.

[24] Samaison L, Uguen A (2022). Idylla MSI test combined with immunohistochemistry is a valuable and cost effective strategy to search for microsatellite instable tumors of noncolorectal origin. Pathol Int.

[25] Schöniger S, Rüschoff J (2022). Mismatch repair deficiency and microsatellite instability. Encyclopedia.

[26] Shia J (2021). The diversity of tumours with microsatellite instability: molecular mechanisms and impact upon microsatellite instability testing and mismatch repair protein immunohistochemistry. Histopathology.

[27] Siemanowski J, Schömig-Markiefka B, Buhl T (2021). Managing difficulties of microsatellite instability testing in endometrial cancer-limitations and advantages of four different PCR based approaches. Cancers.

[28] Smithgall MC, Remotti H, Hsiao SJ (2022). Investigation of discrepant mismatch repair immunohistochemistry and microsatellite instability polymerase chain reaction test results for gynecologic cancers using next-generation sequencing. Hum Pathol.

[29] Stelloo E, Jansen AML, Osse EM (2017). Practical guidance for mismatch repair-deficiency testing in endometrial cancer. Ann. Oncol..

[30] Ukkola I, Nummela P, Pasanen A (2021). Detection of microsatellite instability with Idylla MSI assay in colorectal and endometrial cancer. Virchows Arch.

[31] Wang C, Zhang L, Vakiani E, Shia J (2022). Detecting mismatch repair deficiency in solid neoplasms: immunohistochemistry, microsatellite instability, or both?. Mod Pathol.

[32] Win AK, Jenkins MA, Dowty JG (2017). Prevalence and penetrance of major genes and polygenes for colorectal cancer. Cancer Epidemiol Biomarkers Prev.

[33] Yang RK, Chen H, Roy-Chowdhuri S (2022). Clinical testing for mismatch repair in neoplasms using multiple laboratory methods. Cancers (Basel).

[34] Yoshino T, Pentheroudakis G, Mishima S (2020). JSCOESMO-ASCO-JSMO-TOS: international expert consensus recommendations for tumour-agnostic treatments in patients with solid tumours with microsatellite instability or NTRK fusions. Ann Oncol.

[35] Zhang Q, Young GQ, Yang Z (2020). Pure discrete punctate nuclear staining pattern for MLH1 protein does not represent intact nuclear expression. Int J Surg Pathol.

