# Erratum zu: Testung auf Mismatch-Reparatur-Defizienz und Mikrosatelliteninstabilität

**DOI:** 10.1007/s00292-023-01226-0

**Published:** 2023-09-01

**Authors:** Josef Rüschoff, Hans-Ulrich Schildhaus, Jan Hendrik Rüschoff, Korinna Jöhrens, Tina Bocker-Edmonston, Wolfgang Dietmaier, Hendrik Bläker, Gustavo Baretton, David Horst, Manfred Dietel, Arndt Hartmann, Frederick Klauschen, Sabine Merkelbach-Bruse, Albrecht Stenzinger, Sandra Schöniger, Markus Tiemann, Wilko Weichert, Reinhard Büttner

**Affiliations:** 1 Discovery Life Sciences Biomarker GmbH und Pathologie Nordhessen, Germaniastr. 7, 34119 Kassel, Deutschland; 2 Institut für Pathologie und Molekularpathologie, Universitätsspital Zürich, Zürich, Schweiz; 3 Institut für Pathologie, Universitätsklinikum Carl Gustav Carus Dresden, Dresden, Deutschland; 4 Department of Pathology, Cooper University Health Care, Camden, NJ USA; 5 Institut für Pathologie/Zentrum für molekularpathologische Diagnostik, Universität Regensburg, Regensburg, Deutschland; 6 Institut für Pathologie, Universitätsklinikum Leipzig, Leipzig, Deutschland; 7 Institut für Pathologie, Charité – Universitätsmedizin Berlin, Campus Mitte, Berlin, Deutschland; 8 Pathologisches Institut, Universität Erlangen-Nürnberg, Erlangen, Deutschland; 9 Pathologisches Institut, Ludwig-Maximilians-Universität München, München, Deutschland; 10 Institut für Pathologie, Universitätsklinikum Köln, Köln, Deutschland; 11 Pathologisches Institut, Universitätsklinikum Heidelberg, Heidelberg, Deutschland; 12 Institut für Hämatopathologie Hamburg, Hamburg, Deutschland; 13 Institut für Pathologie, Technische Universität München, München, Deutschland

**Erratum zu:**

**Pathologie 2023**

10.1007/s00292-023-01209-1

In Abb. 1 in diesem Artikel wurde die y‑Achse bei der Anpassung der Abbildung zum Layout falsch interpretiert. Die Abbildung und Legende hätte wie folgt aussehen sollen.

**Figure 1:**
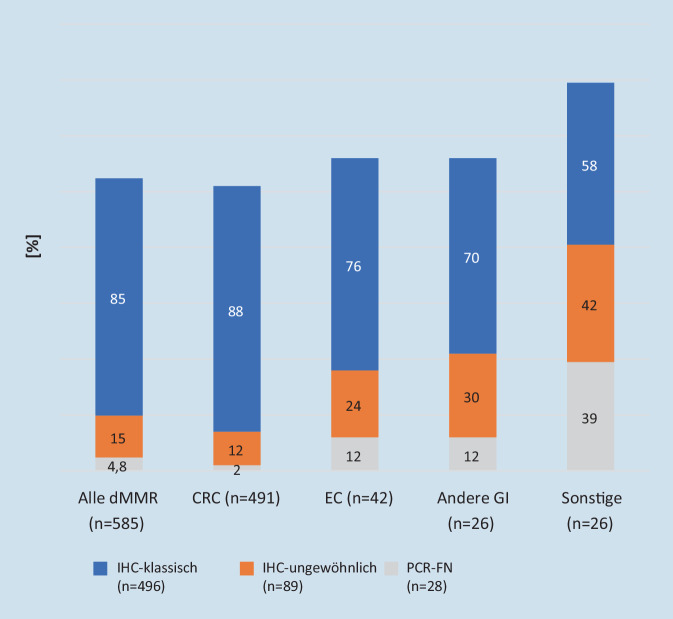

Der Originalbeitrag wurde korrigiert.
