# Supplementary material for: Testing deficient mismatch repair and microsatellite instability: A focused update. German version
Source: Pathologie (Heidelb). 2023 Aug 7;44(5):301–10. [Article in German] doi: 10.1007/s00292-023-01209-1 (PMC10457237; doi:10.1007/s00292-023-01209-1)
Supplement: Supplementary file 1 [file 292_2023_1209_MOESM1_ESM.pdf]

Tab. S1 Häufigkeitsverteilung der unterschiedlichen Befundkonstellationen im französischen Tumorkollektiv (nach [10])

	Klassischer Befund	Ungewöhnlicher dMMR Befund				
	MSI-H & MLH1/PMS2 oder MSH2/MSH6 Verlust n=496	Gesamt n=89	isoliert PMS2 oder MSH6 Verlust Gruppe 1, n=53	dMMR klassisch & MSS/MSI-L Gruppe 2, n=16	pMMR & MSI-H/MSI-L Gruppe 3, n=5	komplexer MMR-Protein Verlust Gruppe 4, n=15
Prävalenz (gesamt)	85%	15%	59,6%	17,9%	5,6%	16,9%
Nicht-CRC	13,1%	32,6%	26,4%	62,5%	20,0%	26,7%
Genetisch/LS	21,4%	44,9%	45,3%	37,5%	53,3%	53,3%
MLH1/PMS2	70-80%	16,8%	Gruppe 2	11	kein MMR Ausfall	5x alle vier
MSH2/MSH6	20-30%	8,9%		5		3x drei
MSH6	s. ungewöhnlich	38,2%	32	Gruppe 1		2x Nicht-Heterodimere
PMS2		28,1%	21			5x heterogen, unklar
